# Protective effect of oral stem cells extracellular vesicles on cardiomyocytes in hypoxia-reperfusion

**DOI:** 10.3389/fcell.2023.1260019

**Published:** 2024-01-15

**Authors:** Ylenia Della Rocca, Francesca Diomede, Fanì Konstantinidou, Oriana Trubiani, Thangavelu Soundara Rajan, Sante D. Pierdomenico, Valentina Gatta, Liborio Stuppia, Guya Diletta Marconi, Jacopo Pizzicannella

**Affiliations:** ^1^ Department of Innovative Technologies in Medicine and Dentistry, University “G. D’Annunzio” Chieti-Pescara, Chieti, Italy; ^2^ Department of Psychological Health and Territorial Sciences, School of Medicine and Health Sciences, “G. D'Annunzio” University of Chieti-Pescara, Chieti, Italy; ^3^ Unit of Molecular Genetics, Center for Advanced Studies and Technology (CAST), “G. D'Annunzio” University of Chieti-Pescara, Chieti, Italy; ^4^ Discovery and Development Department, Hoynoza Technologies Private Limited, Karnataka, India; ^5^ Department of Engineering and Geology, University “G. D’ Annunzio” Chieti-Pescara, Pescara, Italy

**Keywords:** extracellular vesicles, MicroRNAs, human gingival mesenchymal stem cells, cardiomyocytes, acute hypoxia

## Abstract

Hypoxia signaling plays an important role in physiological and pathological conditions. Hypoxia in the heart tissue can produce different consequences depending on the duration of exposure to the hypoxic state. While acute hypoxic exposure leads to a reversible acclimatization in heart tissue with normal systemic oxygen supply, chronic hypoxia exacerbates cardiac dysfunction, leads to a destruction of the tissue. Extracellular vesicles (EVs) are small membrane vesicles that act as mediators of intercellular communication. EVs are secreted by different cell types and those produced by oral cavity-derived mesenchymal stem cells (MSCs), including human gingival MSCs (hGMSCs), have pro-angiogenic and anti-inflammatory effects and showed therapeutic role in tissue regeneration. The aim of the present work was to evaluate the potential protective and regenerative role of EVs produced by hGMSCs, in an *in vitro* model of hypoxia-conditioned HL-1 cardiomyocytes through the expression analysis of following inflammatory, oxidative stress, angiogenesis, cell survival and apoptotic markers: HIF-1α, P300, NFkB, CCL2, IL1B, IL6, NRF2, CASP-3, BAX and VEGF. Results showed that hGMSCs-derived EVs exerted protection HL-1 cardiomyocytes exposed to both pre and post hypoxic conditions. Moreover, modulation of CASP3 and BAX expression demonstrated that EVs reduced the apoptosis. The analysis of microRNAs in EVs derived from hGMSCs was performed to assess the epigenetic regulation of the presented markers. The following microRNAs: hsa-miR-138-5p, hsa-miR-17-5p, hsa-miR-18a-5p, hsa-miR-21-5p, hsa-miR-324-5p, hsa-miR-133a-3p, hsa-miR-150-5p, hsa-miR-199a-5p, hsa-miR-128-3p and hsa-miR-221-3p can directly or indirectly target the studied genes by determining their modulation obtained in our study. The data from this study suggested that EVs obtained from hGMSCs may be considered for the cell free treatment option in hypoxia-driven cardiac tissue dysfunction.

## 1 Introduction

Molecular oxygen is an indispensable component in mammalian cells. In the condition of normal oxygen, mammalian cell consumes oxygen and nutrients to synthesize adenosine 5′-triphosphate (ATP). It is also involved in various key biochemical reactions in the cells. Therefore, mammalian cells keep oxygen balance to assure their physiological function. Decreased oxygen concentration stimulates a variety of downstream signal responses in the cells. In the presence of hypoxic pressure, mammalian cells will activate a series of downstream pathways, mainly including hypoxia-inducible factor (HIF) (Luo et al., 2022). Hypoxia is a condition in which the oxygen levels are lower than the normoxia’s oxygen level. The central pathway of cell response to a low oxygen environment involves HIF transcription factors, which are responsible for sensing the hypoxic environment in the cells, inducing metabolic changes, regulating cell proliferation, and controlling inflammatory response and other functions. HIF-1α transcription factor-mediated hypoxia signaling plays an important role in both physiological and pathological conditions (Della Rocca et al., 2022). At the same time, HIF signal has also displayed the link with different pathologies, such as cardiovascular, metabolic, inflammatory, and infection-related diseases. Tissue oxygenation is essential for the development of an ideal tissue microenvironment or the reconstitution of an altered microenvironment in the damaged tissue followed by hypoxic injury (Darby and Hewitson, 2016). A lower flow of oxygen caused by hypoxic state can be resulted in reversible or irreversible damage to an organ, depending on whether the hypoxic stimulus is of short duration or longer duration, thereby causing not only cell damage but also cell death with other different consequences in the affected tissue (Sies and Jones, 2020). Peripheral blood circulation is required to maintain physiological function in each tissue. Impaired blood circulation decreases oxygen delivery, leading to tissue hypoxia that occurs in several cardiovascular disorders including atherosclerosis, pulmonary arterial hypertension, and heart failure (Abe et al., 2017). Heart failure**,** also known as congestive heart failure (CHF), is a chronic and progressive clinical syndrome induced by structural or functional cardiac abnormalities and caused by an impairment of the heart’s blood pumping function (Arrigo et al., 2020). It is well known that oxidative stress plays an important role in the pathophysiology of cardiac remodeling and also in heart failure, since it determines molecular alterations of the intracellular pathways, causing cellular dysfunction (Tsutsui et al., 2011).

Formation of reactive oxygen species (ROS), nitric oxide (NO) and superoxide through NADPH in cardiac tissue may lead to mitochondrial damage, alteration of cardiac contractility and formation of atherosclerosis (Balligand et al., 1992; Babior, 2000). Recent studies indicated that hypoxia may play a key role in the progression of atherosclerotic plaques through lipid accumulation, ATP depletion and angiogenesis, which produce advanced lesions (Hulten and Levin, 2009).

Progression of heart diseases are sustained by the loss of cardiomyocyte. Cardiomyocytes damage can be caused by hypoxia state that induces oxidative stress and apoptosis (Giordano, 2005).

HIF has an impact in several cardiac phenotypes including heart failure (Murry et al., 1986). Following acute hypoxic exposure, the heart tissue responds with a reversible acclimatization due to an immediate increase in heart rate and an increase in lung function that allow maintaining optimal oxygen supply at the systemic level (Lu et al., 2015; Pearson, 2015).

Cardiovascular diseases are one of the main causes of death worldwide. Treatment through regenerative medicine approaches has emerged as a new platform for heart failure (Garbern and Lee, 2022). In particular, the use of extracellular vesicles (EVs) gained a lot of interest in the scientific community (Doyle and Wang, 2019).

EVs are natural nanoparticles containing biologically active molecules like lipids, proteins and different types of nucleic acids, which are enclosed by lipid membrane (Gupta et al., 2021).

EVs have been isolated from most bodily fluids and in the last years it is becoming increasingly clear that they have a central role not only in the regulation of normal physiological events, such as stem cell maintenance, tissue repair, immune surveillance and blood coagulation (Yanez-Mo et al., 2015). Accordingly, they represent important mediators of intercellular communication in both physiological and pathological conditions (Raposo and Stoorvogel, 2013; Wiklander et al., 2019).

EVs are released by almost all cell types which contribute to the correct intercellular communication, mediating the biological effects by transferring proteins, lipids, and nucleic acids (EL Andaloussi et al., 2013).

The most studied molecules included in the EVs content are the microRNAs (miRNAs). miRNAs are small non-coding RNAs which perform post-transcriptional regulation. Since the expression profile of miRNAs has specific characteristics for each physiological or pathological tissue condition, they could serve as pathological biomarkers and have therapeutic action (Liu et al., 2019).

Furthermore, recent data indicate that EVs can also be investigated directly as promising therapeutic agents for tissue repair and immune response modulation. For instance, EVs from mesenchymal stem cells (MSCs) have been utilized to induce tissue regeneration following myocardial infarction (Lai et al., 2011; Timmers et al., 2011).

EVs isolated from oral cavity-derived mesenchymal stem cells (MSCs) have shown to possess pro-angiogenic and anti-inflammatory effects, which contribute for their potential role in regenerative medicine (Diomede et al., 2022; Fonticoli et al., 2022). Indeed, EVs can be used as an alternative therapy for tissue regeneration either in their native form or as vehicles for the delivery of other therapeutic agents since they are able to cross biological barriers while also protecting the contents from degradation (Imafuku and Sjoqvist, 2021).

MSCs are largely described as multipotent non hematopoietic adult stem cells that showed a positive expression for CD73, CD90, and CD105, surface markers, and a negative expression for hematopoietic markers as CD14, CD34, and CD45 (Teixeira et al., 2013; Pagella et al., 2021). They can be isolated from different adult tissues, including oral cavity tissues as dental pulp, periodontal ligament and gingiva. MSCs showed some important features, like differentiation ability, self-renewal potential and immunomodulatory properties. To date the MSCs are considered a promising tool to promote the tissue regeneration in diverse pathological conditions. MSCs are able to migrate to the damaged site, engrafting, and subsequently differentiating into desired cells for tissue regeneration. Latest research demonstrated that the EVs are considered the key mechanism to release the biological factor and regulate the intercellular communication of MSCs action (Keshtkar et al., 2018; Harrell et al., 2019).

De Castro et al. reported that the transplantation of EVs derived from MSCs showed a therapeutic effect as well as the use of MSCs in asthma disease model (de Castro et al., 2017). The use of EVs is considered more advantageous than the transplantation of whole cells. EVs are easy to isolate, store and they showed less side effect in the *in vivo* study other than they maintain their biological properties (Chiricosta et al., 2020; Silvestro et al., 2020). Thus, the MSCs secrete factors represent a promising therapeutic strategy for use in the regenerative medicine clinical approach (Lai et al., 2015; Wu et al., 2020).

The present work aimed at evaluating the potential protective and regenerative role of EVs produced by human gingival MSCs (hGMSCs) against acute hypoxia in an *in vitro* model of HL-1 cardiomyocytes through the modulation of HIF-1α, P300, Nuclear Factor kappa B (NFkB), C-C Motif Chemokine Ligand 2 (CCL2), Interleukin 1 beta (IL1B), Interleukin 6 (IL6), nuclear factor erythroid 2–related factor 2 (NRF2), Caspase-3 (CASP3), Bcl-2-associated X protein (BAX) and Vascular Endothelial Growth Factor (VEGF), also from the point of view of the epigenetic modulation. In EVs derived from hGMSCs we have identified the following microRNAs: hsa-miR-138-5p, hsa-miR-17-5p, hsa-miR-18a-5p, hsa-miR-21-5p, hsa-miR-324-5p, hsa-miR-133a-3p, hsa-miR-150-5p, hsa-miR-199a-5p, hsa-miR-128-3p and hsa-miR-221-3p. These miRNAs may target some of the analyzed inflammatory, oxidative stress, angiogenesis, cell survival and apoptotic markers.

## 2 Materials and methods

### 2.1 Cell culture

HL-1 cells (Sigma-Aldrich, Milan, Italy) were maintained in Claycomb medium completed with 10% fetal bovine serum (FBS, Euroclone, Milan, Italy), 2 mML-glutamine, 0.1 mM norepirephrine, and 100 μg/mL penicillin/streptomycin (Lonza, Basel, Switzerland). The cells were maintained at 37 °C in a humidified atmosphere of 5% of CO_2_ in air and subcultured until they reached 80% confluence (Marconi et al., 2022). Human gingival mesenchymal stem cells (hGMSCs) were isolated from gingival tissues of three patients, in good general health conditions and without oral disease, who underwent surgical procedure. The gingival tissues were placed in a culture dish, fragmented, washed several times in phosphate-buffered saline solution (PBS, Lonza, Basel, Switzerland) with 5% of gentamicin (Lonza) and transferred in the incubator at 37°C in a humidified atmosphere of 5% CO2 in air with mesenchymal stem cell growth medium-chemically defined (MSCGM-CD, Lonza). The medium was replaced every 2 days for approximately 2 weeks before cells reached 80% confluence and were passaged in culture (Marconi et al., 2021c).

### 2.2 Hypoxic culture

The HL-1 cells were cultured in Claycomb medium com-pleted with 10% fetal bovine serum (Euroclone, Milan, Italy), 2 mM L-glutamine, 0.1 mM norepirephrine, and 100 μg/mL penicillin/streptomycin (Lonza, Basel, Switzerland) under 0.2% hypoxia for 24 h using ProOx Model P110 (BioSpherix, 25 Union Street, Parish, NY 13131) hypoxia chamber as per the referred manual of ProOx Model P110. In CTRL sample, after 24 h of acute hypoxia stimulus, the cells were successively cultured in normoxia for the next 24 h at 37°C in a humidified atmosphere of 5% of CO_**2**_ in air. In the sample entitled HL-1 + EVs-hGMSCs UN, the HL-1 cells were maintained under acute hypoxic stimulus at 0.2% for 24 h and then treated with EVs derived from hGMSCs under normoxia for 24 h. In the sample entitled HL-1 + EVs-hGMSCs UH, the HL-1 were first treated with EVs derived from hGMSCs and kept in hypoxia at 0.2% for 24 h and then in normoxia for 24 h.

### 2.3 EVs isolation

Human GMSCs were seeded at confluence in 150 mm cell culture dishes. After 48 h of culture, EVs were isolated from the conditioned medium of hGMSCs. For EV extraction, the ExoQuick TC commercial agglutinant (System Biosciences, Euroclone SpA, Milan, Italy) was utilized. Briefly, 2 mL of ExoQuick TC solution was added to 10 mL of conditioned medium. The mix was incubated overnight at 4 °C without rotation; one centrifugation step was executed at 1,500× *g* for 30 min to sediment the EVs, and the pellets were re-suspended in 200 μL of Phosphate buffered saline (PBS). The EVs used in the current paper were isolated and characterized trough Western blotting as described by Pizzicannella et al. (Pizzicannella et al., 2019), from which data is evident EVs positivity for CD9, CD63 and CD81.

### 2.4 Experimental study design

The study design is reported as follows:

CTRL: HL-1 cells were kept in hypoxia at 0.2% for 24 h and successively in normoxia for 24 h.

HL-1 + EVs-hGMSCs UN: HL-1 were kept in hypoxia at 0.2% for 24 h and then treated with hGMSCs EVs under normoxic stimulus for successive 24 h.

HL-1 + EVs-hGMSCs UH: HL-1 treated with hGMSCs EVs were kept in hypoxia at 0.2% for 24 h and then in normoxia for successive 24 h.

### 2.5 Confocal microscopy analysis

The HL-1 cells were seeded at 8,500/well on 8-well culture glass slides (Corning, Glendale, Arizona, United States) under hypoxia state for 24 h and treated with hGMSCs EVs both in EVs post hypoxia and EVs pre hypoxia conditions and processed for immunofluorescence analyses as previously described (Trubiani et al., 2012a). Then, the samples were fixed for 1 h with 4% paraformaldehyde in 0.1 M PBS (pH 7.4) (Lonza, Basel, Switzerland) at room temperature. After washing, cell samples were processed for the immunofluorescence staining: cells were permeabilized with 0.5% Triton X-100 in PBS (Lonza) for 10 min and blocked with 5% skimmed milk in PBS for 1 h. The HL-1 cells were incubated with the primary antibodies for1 h at room temperature using mouse monoclonal antibodies: HIF-1α (1:200) (sc-53546, Santa Cruz Biotechnology, Dallas, TX, United States), NFkB p65 (1:200) (sc-8008, Santa Cruz Biotechnology), p300 (1:200) (sc-32244, Santa Cruz Biotechnology), NRF2 (1:200) (sc-365949, Santa Cruz Biotechnology), VEGF (1:200) (sc-57496, Santa Cruz Biotechnology), and CASP3 (1:200) (sc-56052, Santa Cruz Biotechnology) (Diomede et al., 2019). Then, the HL-1 cells were incubated with secondary antibody Alexa Fluor 568 red fluorescence conjugated goat anti-mouse antibody (1:200; Molecular Probes, Invitrogen, Eugene, OR, United States), for 1 h at 1 h, 37°C; successively, the cells were incubated with Alexa Fluor 488 phalloidin green fluorescent conjugate (1:200; Molecular Probes), TOPRO (1:200; Molecular Probes) (1 h, 37°C). The Zeiss LSM800 confocal system (Carl Zeiss, Jena, Germany) was used to acquire microphotographs. The experiment was conducted in triplicate.

### 2.6 Western blotting analysis

The proteins derived from HL-1 cell cultures of the three experimental points were used at the concentration of 50 μg for the electrophoresis and subsequent transfer on the membrane of polyvinylidene fluoride (PVDF) as previously described (Trubiani et al., 2012b). Successively, they were blocked in 5% of non-fat milk in PBS +0.1% Tween-20. Then, the blotted membranes were incubated overnight at 4°C with the primary antibodies of HIF-1α (1:500) (sc-53546, Santa Cruz Biotechnology), NFkB p65 (1:500) (sc-8008, Santa Cruz Biotechnology), p300 (1:500) (sc-32244, Santa Cruz Biotechnology), NRF2 (1:500) (sc-365949, Santa Cruz Biotechnology), VEGF (1:500) (sc-57496, Santa Cruz Biotechnology), and CASP3 (1:500) (sc-56052, Santa Cruz Biotechnology). GAPDH was used as loading control (1:750) (sc-69879, Santa Cruz Biotechnology). After washes in PBS containing 0.1% Tween-20, membranes were incubated for 1 h at room temperature with peroxidase-conjugated anti-mouse secondary antibody (A90-116 P Goat anti-mouse; Invitrogen; 1:5000 dilution in 2.5% milk made by 1X PBS and 0.1% Tween-20). The enhanced chemiluminescence detection method (ECL) was used to visualize protein expression through UVIband-1D gel analysis (Uvitec, Cambridge, United Kingdom) with photo documenter Alliance 2.7 (Uvitec, Cambridge, United Kingdom). The data obtained were normalized with GAPDH loading control. The experiment was conducted in triplicate.

### 2.7 Inflammation and apoptotic markers expression analysis in real-time PCR

The inflammatory markers expression *IL1B*, *CCL2*, *IL6* and apoptotic-related genes *BAX* and *CASP3* were analyzed by Real-Time PCR. Total RNA was extracted using PureLink RNA Mini Kit (Ambion, Thermo Fisher Scientific, Milan, Italy) according to the manufacturer’s instructions. Three independent biological replicates were analyzed for each sample. Two micrograms of total RNA was retrotranscribed using M-MLV Reverse Transcriptase (M1302 Sigma-Aldrich) to synthesize cDNA for 10 min at 70 °C, 50 min at 37 °C and 10 min at 90 °C according to the technical bulletin. Real-Time PCR was performed with Mastercycler ep real plex Real-Time PCR system (Eppendorf, Hamburg, Germany). The levels of mRNA expression of *IL1B, CCL2, IL6,BAX, CASP3* and *GAPDH* (endogenous marker) were evaluated in CTRL, HL-1 + EVs-hGMSCs UN, and HL-1 + EVs-hGMSCs UH conditions. Commercially available PrimeTime™ Predesigned qPCR Assays of *IL1B* (Hs.PT.58.1518186, Tema Ricerca Srl, Castenaso, Italy); *CCL2* (Hs.PT.58.45467977, Tema Ricerca Srl); *IL6* (Hs.PT.58.40226675, Tema Ricerca Srl); *BAX* (Hs.PT.56a.19141193. g, Tema Ricerca Srl); *CASP3* (Hs.PT.56a.25882379. g, Tema Ricerca Srl); *GAPDH* (Hs.PT.39a.22214836, Tema Ricerca Srl, Castenaso, Italy); and the PrimeTime™ Gene Expression Master Mix (cat.n°1055772, Tema Ricerca Srl) were utilized according to the protocol (Marconi, 2021a). Expression levels for each gene were performed according to the 2^−ΔΔCT^ method. Real-Time PCR was performed in three independent experiments.

### 2.8 RNA extraction from hGMSCs-derived EVs

Total RNA, including microRNAs (miRNAs), was manually isolated from EVs of hGMSCs using the Total Exosome RNA and Protein Isolation Kit (Invitrogen, Thermo Fisher Scientific, Waltham, MA, United States) according to the manufacturer’s instructions. Quantity and quality of total RNA were assessed by microvolume UV–vis spectrophotometer NanoPhotometer (Implen, GmbH, Munich, Germany) (Marconi et al., 2021b).

### 2.9 MicroRNAs’ characterisation profile in hGMSCs-derived EVs

MicroRNA detection analysis in EVs retrieved from hGMSCs was carried out by TaqMan™ Array Human MicroRNA A Cards v2.0 (Cat. 4398965, Applied Biosystems**,** Foster City, CA, United States). An initial Megaplex RT Reaction was performed in order to reverse-transcribe miRNAs, using the Megaplex RT primer Pool A and TaqMan MicroRNA Reverse Transcription kit (Applied Biosystems, Waltham, MA). Afterwards, a preamplification reaction of complementary DNAs (cDNAs) was also executed through use of the TaqMan PreAmp Master Mix 2X and Megaplex PreAmp Primers pool A (Applied Biosystems, Waltham, MA). The preamplified products were then diluted with 0.1X TE at pH 8.0 and subsequently mixed with the TaqMan™ universal Master Mix II, no UNG (Applied Biosystems, Waltham, MA) and nuclease-free water. Finally, they were loaded into the TaqMan™ Array Human MicroRNA A plates and processed by qRT- PCR, using a QuantStudioTM 7 Pro Real-Time PCR detection system (Life Technologies, Carlsbad, CA, United States).) Solely a characterization analysis of the small non-coding RNAs was carried out, taking into consideration exclusively amplification cycles (Cts) < 35 of detected miRNAs (Supplementary Figure S3). The TaqMan Array Human MicroRNA A Card v2.0 contains 384 TaqMan MicroRNA Assays of the most highly characterized miRNAs in humans, enabling accurate quantitation of 377 human microRNAs through Real-Time PCR technology. Three TaqMan MicroRNA Assay endogenous controls are included to aid in data normalization and one TaqMan MicroRNA Assay not related to human is included as a negative control.

### 2.10 IPA functional and biological network analysis

Characterised miRNAs were analysed by Ingenuity Pathway Analysis (IPA) software in order to highlight the principal functions, cellular processes and molecular networks in which they actively participate. IPA-inferred network analysis was generated for the miRNAs and genes of interest *HIF1Α*, *P300* (also known as *EP300*), *NFkB* (*NFkB1*), *NRF2* (also known as *NFE2L2*), *VEGF* (*VEGFA*) and caspase 3 (CASP3)associating their functionality with the function of other small non-coding RNAs and genes, as well as with each other. The mechanistic network of these molecules was evidenced based on their connectivity and enrichment statistics.

### 2.11 Statistical analysis

One-Way ANOVA test and *post hoc* Tukey’s multiple comparisons test were performed through GraphPad Prism 5 software to evaluate the statistical differences. The value of *p* < 0.05 was set as a statistically significant value.

## 3 Results

### 3.1 hGMSCs–derived EVs reduced the expression of hypoxia and pro-inflammatory markers

**FIGURE 1:**
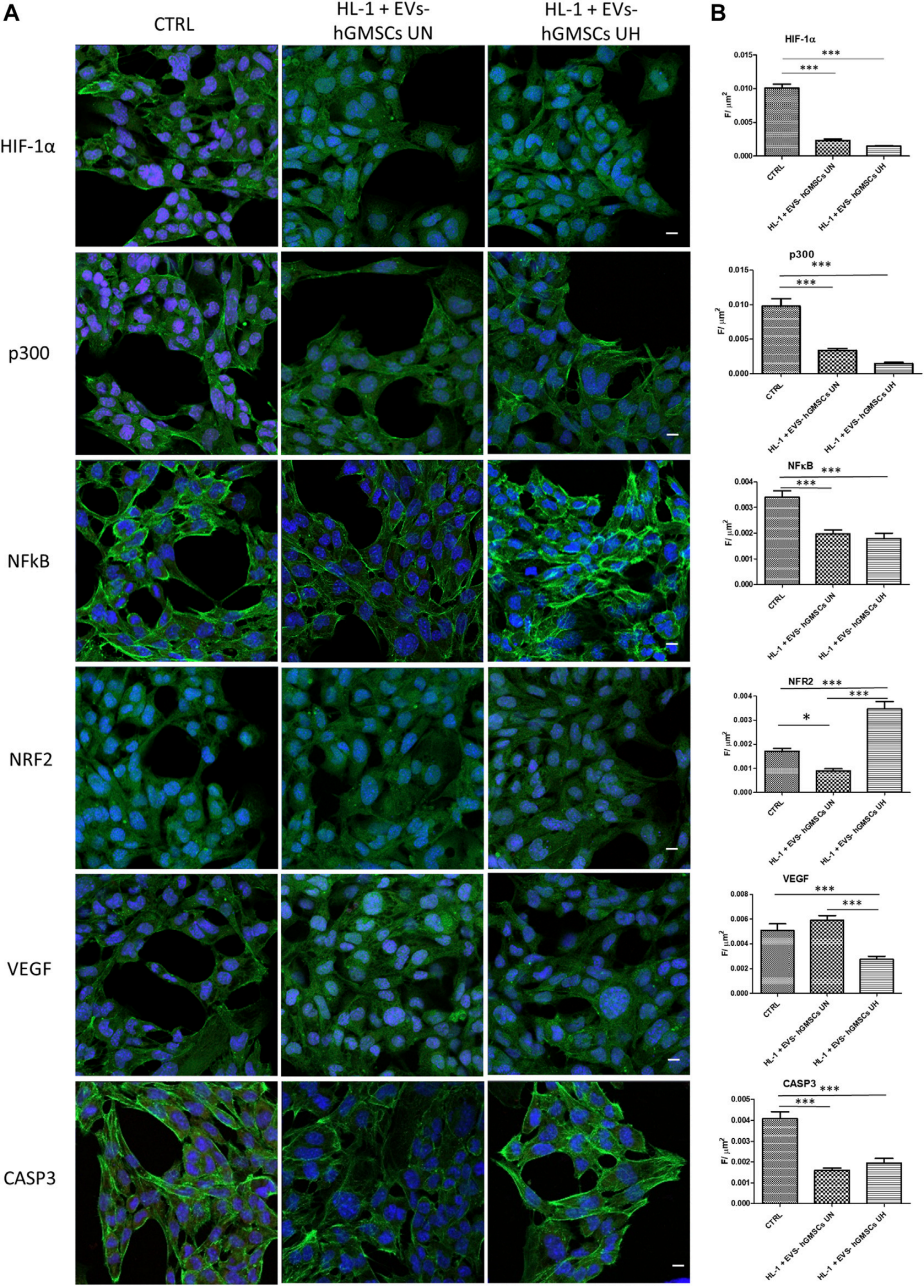
**(A)** Immunofluorescence analysis of HIF-1α, p300, NFkB, NRF2, VEGF, and CASP3 expression in CTRL, HL-1 + EVs-hGMSCs UN and HL-1 + EVs-hGMSCs UH. MERGE (red fluorescence: markers; Green fluorescence: cytoskeleton actin; blue fluorescence TOPRO: nuclei. Scale bar: 10 μm. **(B)** CLSM quantitative analysis of HIF-1α, p300, NFkB, NRF2, VEGF, CASP3 expression calculated as arbitrary unit of fluorescence per cell surface unit ((F/μm^2^). Data are expressed as mean ± S.E.M. *p < 0.05; **p < 0.01; ***p < 0.001. Statistical analysis was performed by one-way ANOVA and a *post hoc* Tukey’s multiple comparison analysis.

The immunofluorescence showed that the levels of hypoxia markers HIF-1α and P300 were significantly downregulated in EVs-hGMSCs UN and HL-1 + EVs-hGMSCs UH compared to the CTRL sample with greater evidence of HIF-1α decreased in EVs-hGMSCs UN (Figure 1). Moreover, inflammatory marker NFkB was found to be reduced in EVs-hGMSCs UN and HL-1 + EVs-hGMSCs UH compared to the CTRL sample (Figure 1).

The real-time evidenced that IL1B was found to be upregulated in CTRL compared to HL-1 + EVs-hGMSCs UN and HL-1 + EVs-hGMSCs UH samples. The gene expression of IL1B in HL-1 + EVs-hGMSCs UN showed a significant higher expression of IL1B compared to HL-1 + EVs-hGMSCs UH (Figure 3).

Moreover, the CCL2 gene expression was significant higher in CTRL compared to EVs-hGMSCs UN and EVs-hGMSCs UH. The CCL2 gene expression was higher in EVs-hGMSCs UH compared to EVs-hGMSCs UN (Figure 3).

In addition, the gene expression of IL6 was also evaluated evidencing a significant upregulation of the IL6 gene expression in CTRL compared to HL-1 + EVs-hGMSCs UN and HL-1 + EVs-hGMSCs UH samples. Additionally, the gene expression of IL6 in HL-1 + EVs-hGMSCs UN showed a significant higher expression of IL6 compared to HL-1 + EVs-hGMSCs UH (Figure 3).

### 3.2 hGMSCs–derived EVs enhanced the expression of antioxidant and angiogenic markers and decreased CASP3 and BAX apoptotic markers

The level of NRF2 immunofluorescence was found higher in HL-1 + EVs-hGMSCs UH compared to HL-1 + EVs-hGMSCs UN and CTRL sample (Figure 1). Similarly, VEGF, an angiogenic factor, was found to be significant overexpressed in HL-1 + EVs-hGMSCs UN compared to the CTRL and HL-1 + EVs-hGMSCs UH (Figure 1). CASP3 level expression showed a significant reduction in both HL-1 + EVs-hGMSCs UN and HL-1 + EVs-hGMSCs UH compared to CTRL (Figure 1).

**FIGURE 2:**
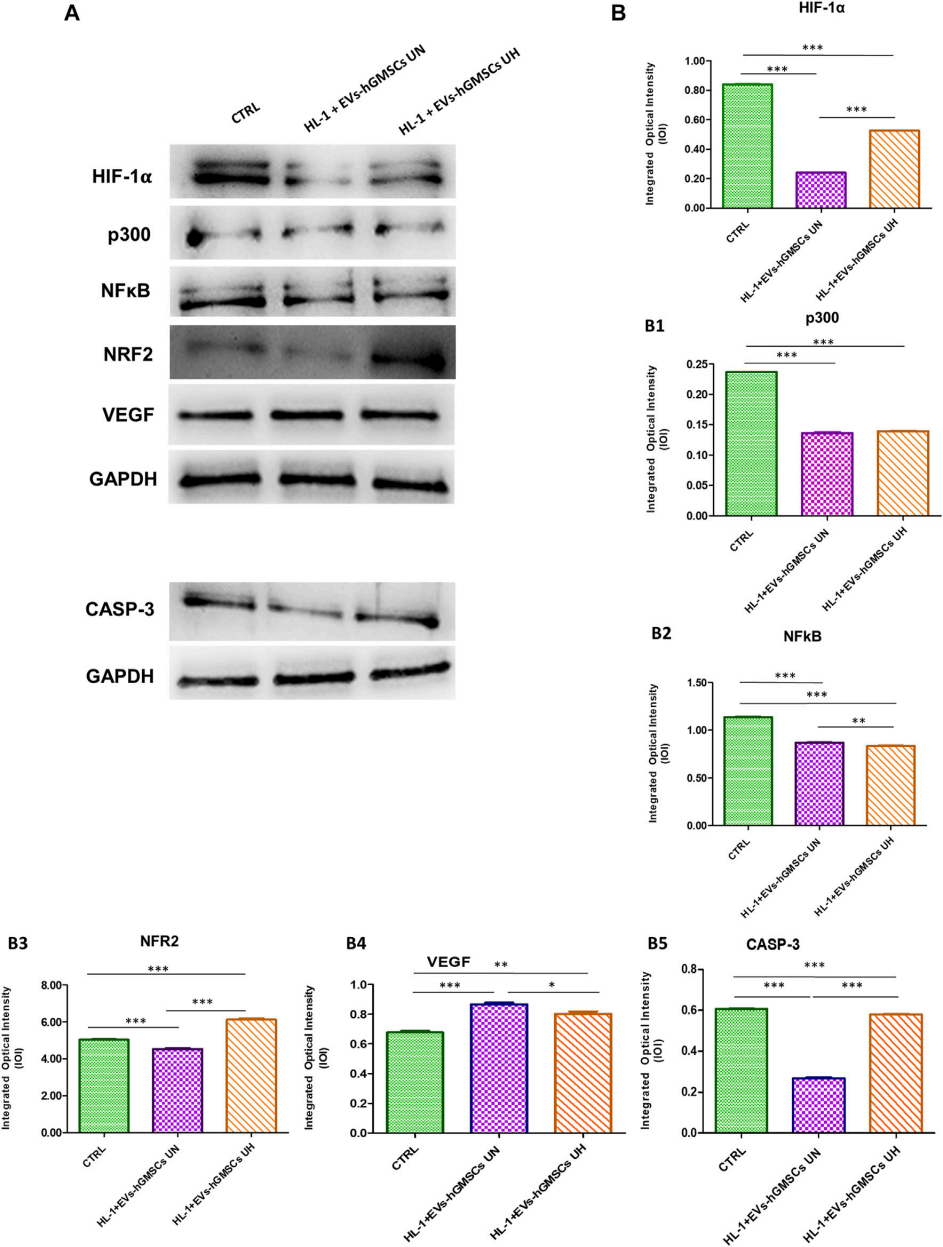
HIF-1α, p300, NFkB, NRF2, VEGF and CASP3 protein expression in CTRL, HL-1 + EVs-hGMSCs UN and HL-1 + EVs-hGMSCs analyzed by Western blotting. Each membrane was probed with GAPDH antibody to verify the loading consistency. Western blot data is representative of three different experiments. **(A)** Western blotting-specific bands of HIF-1α, p300, NFkB, NRF2, VEGF and CASP3 in CTRL, HL-1 + EVs-hGMSCs UN andHL-1 + EVs-hGMSCs UH. [**(B)**-(B5)] Histograms represent densitometric measurements of proteins bands expressed as the integrated optical intensity (IOI) mean of three separate experiments. The error bars show the standard deviation (±SD). Densitometric values analyzed by ANOVA (*post hoc* application of Tukey’s multiple comparison test) returned significant differences. *p < 0.05; **p < 0.01; ***p < 0.001.

The data obtained by Western blotting analysis to evaluate the protein expressions of HIF-1α, P300, NFkB, NRF2, VEGF and CASP3 confirmed the results obtained by Confocal Microscopy (Figure 2).

**FIGURE 3:**
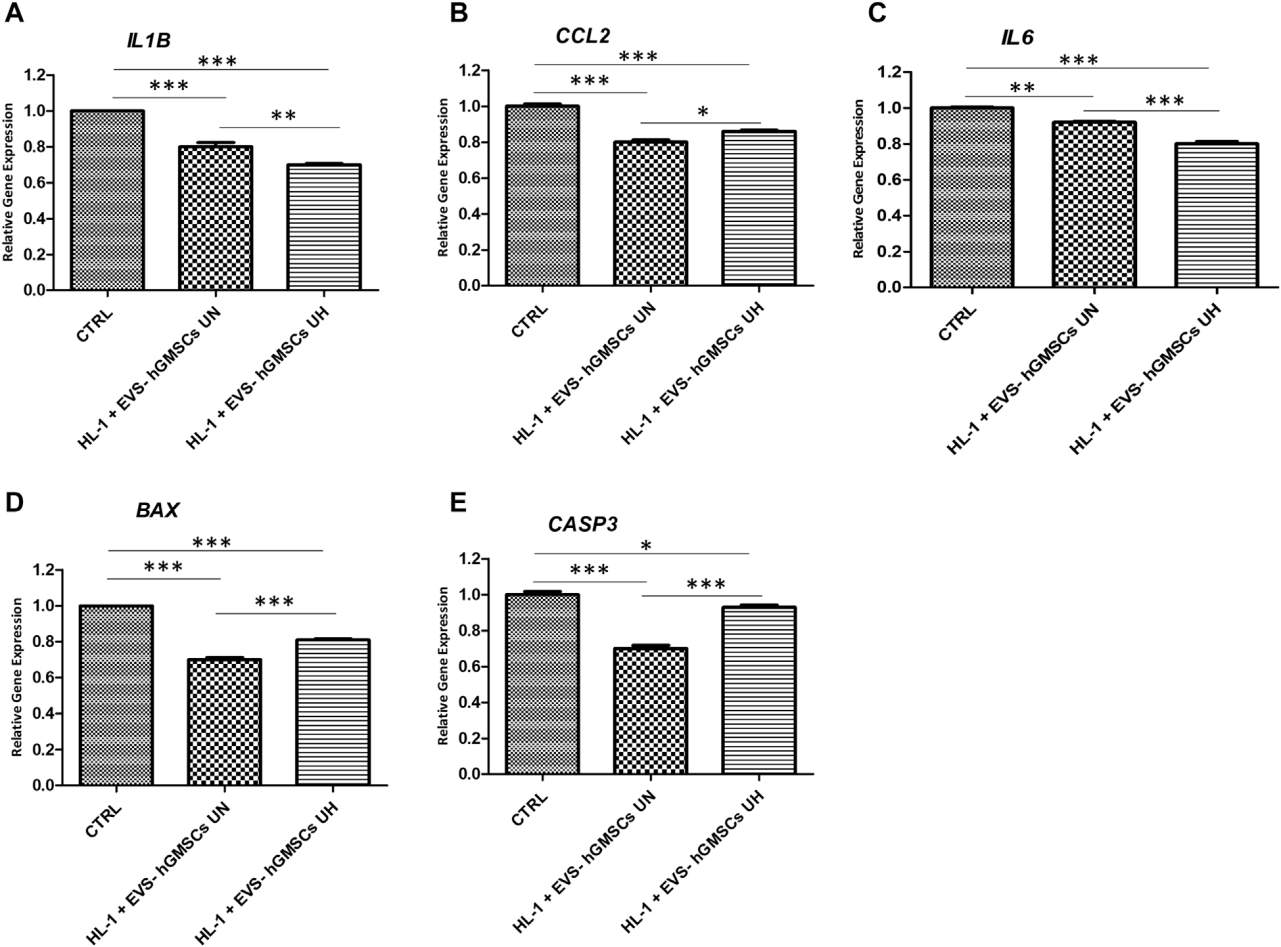
**(A–E)** Histograms of real-time PCR for IL1B, CCL2, IL6, BAX and CASP3 related gene expression in CTRL, HL-1 + EVs-hGMSCs UN and HL-1 + EVs-hGMSCs UH. *p < 0.05; **p < 0.01; ***p < 0.001.

The CASP3 results obtained by CLSM analysis were confirmed by qPCR analysis. The real-time PCR data for BAX gene expression analysis showed a significant downexpression of this marker in HL-1 + EVs-hGMSCs UN and HL-1 + EVs-hGMSCs UH compared to the CTRL sample. BAX gene expression was found to be significant downregulated in HL-1 + EVs-hGMSCs UN compared to HL-1 + EVs-hGMSCs UH. (Figure 3).

### 3.3 MicroRNAs in hGMSCs–derived EVs are predicted to target inflammatory, oxidative stress, angiogenesis, cell survival and apoptotic markers

**FIGURE 4:**
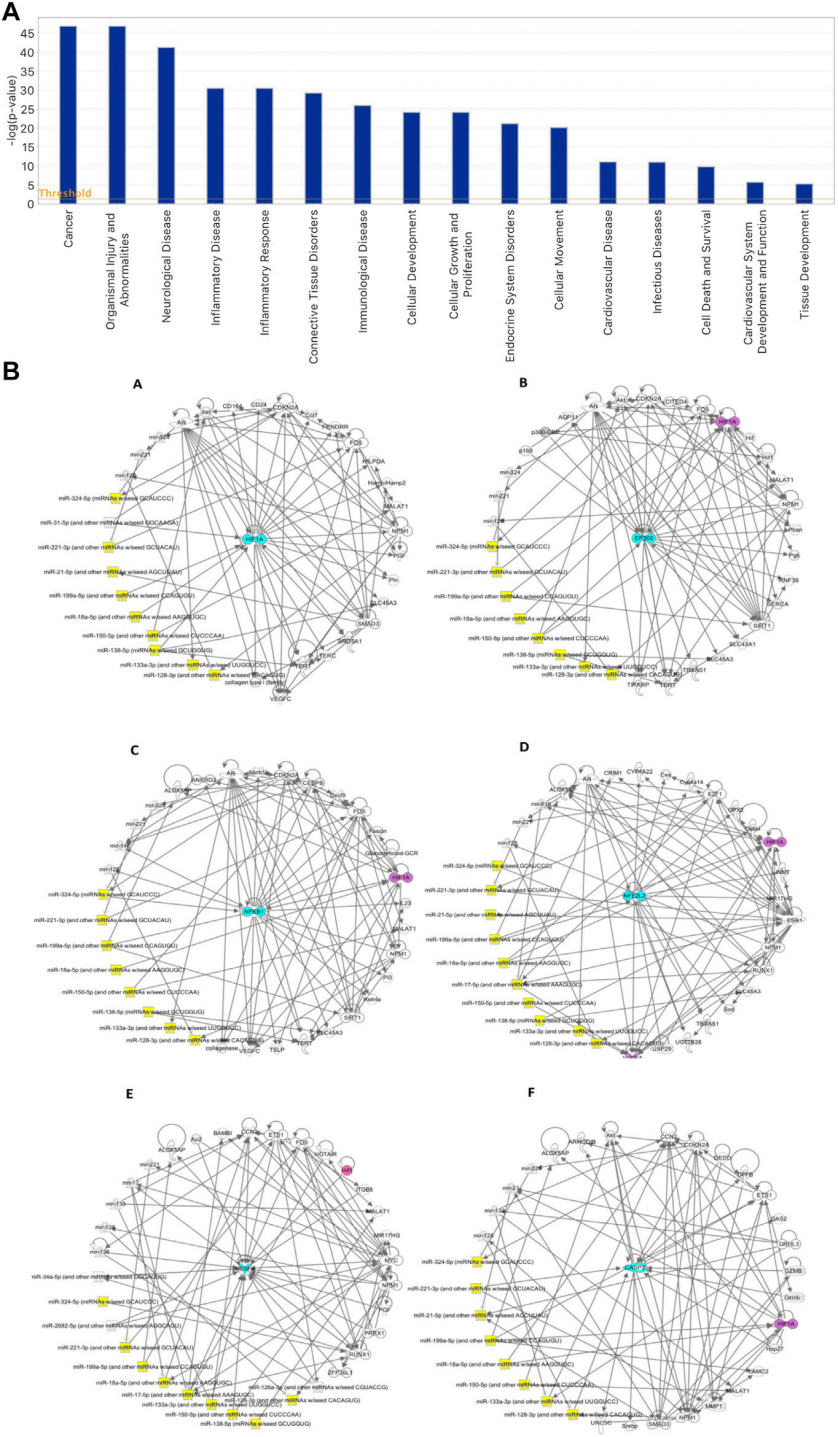
**(A)** IPA-generated bar chart of the principal biological functions modulated by the characterised microRNAs in hGMSCs–derived EVs. **(B)** IPA-inferred miRNAs and target genes networks in EVs isolated from hGMSCs. The networks are centered around 6 key node genes, which are known inflammatory, oxidative stress, angiogenesis, cell survival and apoptotic mediators. The central nodes in question are represented in turquoise by the markers (A) *HIF1Α*, (B) *EP300*, (C) *NFkB1*, (D) *NFE2L2*, (E) *VEGFA* and (F) *CASP3*. The 10 miRNAs of greater interest, based on their main biological functions, on the other hand, are provided by hsa-miR-138-5p, hsa-miR-17-5p, hsa-miR-18a-5p, hsa-miR-21-5p, hsa-miR-324-5p, hsa-miR-133a-3p, hsa-miR-150-5p, hsa-miR-199a-5p, hsa-miR-128-3p and hsa-miR-221-3p and are shown in yellow. In purple, *HIF1Α* is indicated, having been found present in some of the other networks centered around the remaining markers.

Based on the IPA functional analysis, characterised miRNAs in our cohort of hGMSCs–derived EVs have been found to be involved in processes such as inflammatory response, cellular growth and proliferation, cardiovascular system development and function, as well as cardiovascular disease, cell death and survival and tissue development (Figure 4). A search of existing literature further contributed to select a subgroup of the most experimentally important miRNAs detected, coming up with the following 10 microRNAs, hsa-miR-138-5p (MIMAT0000430, miRBase), hsa-miR-17-5p (MIMAT0000070, miRBase), hsa-miR-18a-5p (MIMAT0000072, miRBase), hsa-miR-21-5p (MIMAT0000076, miRBase), hsa-miR-324-5p (MIMAT0000761, miRBase), hsa-miR-133a-3p (MIMAT0000427, miRBase), hsa-miR-150-5p (MI0000479, miRBase), hsa-miR-199a-5p (MI0000242, miRBase), hsa-miR-128-3p (MIMAT0000424, miRBase) and hsa-miR-221-3p (MIMAT0000278, miRBase). Alongside a further qualitative analysis performed by the IPA software, more light was shed on the networks in which these microRNAs and analysed markers *HIF-1α*, *P300* (*EP300*), *NFkB* (*NFkB1*), *NRF2* (*NFE2L2*), *VEGF* (*VEGFA*) and *CASP3* are involved, as well as on their reciprocal relationship from a molecular point of view. As a result, it emerged that the reported 10 miRNAs can target directly or indirectly these genes, potentially silencing their expression and indicating the presence of a possible underlying epigenetic mechanism associated with their modulation. Top networks generated by IPA are provided in Figure 4 and are centered around the genes in question.

## 4 Discussion

The heart needs oxygen to maintain effective contractility. Hypoxia inducible factor-1α (HIF-1α) controls oxygen supply, regulating angiogenesis, vascular remodeling and oxygen utilization, by controlling glucose metabolism and redox homeostasis. Previous work reported that in an *in vivo* study HIF-1, a transcription factor that functions as a master regulator of oxygen homeostasis, plays a fundamental role in the pathophysiology of ischemic heart disease and heart failure (Burgueno et al., 2013).

The present work analyzed the effect of the EVs produced by hGMSCs in cardiac patients under acute hypoxia stimulus. In detail, the HL-1 cells were maintained under acute hypoxic stimulus and then treated with EVs derived by hGMSCs under normoxia (HL-1 + EVs-hGMSCs UN) or the cells were treated with EVs derived by hGMSCs and kept in hypoxia and then in normoxia (HL-1 + EVs-hGMSCs UH). HIF-1α, stabilized under hypoxic conditions (Salceda and Caro, 1997), decreased with EVs treatment both in HL-1 + EVs-hGMSCs UH and HL-1 + EVs-hGMSCs UN suggesting a protective effect of EVs against hypoxia. The potential protective role of EVs derived by hGMSCs was further confirmed by the results obtained of p300 expression) which is a coactivator of HIF-α (Yu et al., 2020). Moreover, hypoxia-triggered oxidants activate the expression of NRF2 transcription factor that protects the cells against damage by controlling numerous downstream genes (Kensler et al., 2007). Interestingly, our data evidenced that EVs derived by hGMSCs increased NRF2 protein expression especially in HL-1 treated with EVs-hGMSCs UH, demonstrating their protective role against oxidative stress. The key transcription factor involved in the inflammation process is NFkB (Nennig and Schank, 2017). The chemokine CCL2 and the cytokines IL1B and IL6 are other proteins involved in inflammation (Ashton et al., 2013). The hypoxia can upregulate CCL2 in a HIF-1-dependent manner, since CCL2 in the promoter region has a HIF-1 binding motif (Stowe et al., 2012). Tissue injury is characterized by an upregulation in the gene expression of cytokines IL-6, IL-8, and chemokines CCL2, CXCL1, CXCL2. IL-6 induces the chemokines CCL2 and IL-6 secretion is determinated by many other inflammatory mediators including IL-1β (Wang et al., 2009). Our data showed NFkB, CCL2, IL1B and IL6 downexpression in treated samples UN and UH, that might indicate the potential anti-inflammatory properties of EVs derived by hGMSCs. In addition, our evidences reported an increase expression of VEGF, a key regulator of physiological angiogenesis, when the cells where treated with EVs exposed to acute hypoxic stimulus, which can promote the angiogenic process via its pro-angiogenic activity (Melincovici et al., 2018). Taken together these results may indicate a potential regenerative role of EVs derived by hGMSCs against acute hypoxia.

In parallel, cell apoptosis process was also investigated through the analysis of CASP3 and BAX expression.

The human caspase family is distributed into three principal groups, largely based on sequence likeness and biological properties. Group one includes the inflammatory such as caspases-1, -4, and -5 based on the commonalities of having a long caspase-recruitment domain and a preference for a large aromatic or hydrophobic residue at the P4 position. The Group two included the apoptotic effector such as caspases-3, -6, and -7 that share a similar short pro-domain, and are named ‘executors of apoptosis’. Among them, CASP3 is a frequently activated death protease, catalyzing the specific cleavage of many key cellular proteins (Lamkanfi et al., 2007). The B cell lymphoma 2 (Bcl-2) family plays a key role in the intrinsic apoptotic pathway triggered by mitochondrial dysfunction in the Bcl-2/Bax/Cleaved CASP3 signaling pathway regulating cell apoptosis and survival processes; CASP3 activation plays a key role to induce the apoptosis following genotoxic stress (Choudhary et al., 2015; Zhang et al., 2019). *BAX* gene is a member of the Bcl-2 gene family that has a pro-apoptotic effect in the intrinsic apoptosis regulation, inducing the release of cytochrome C from mitochondria and the cell death (Pawlowski and Kraft, 2000). CASP3 and BAX are directly related since BAX/BAK signaling activates the effector caspases caspase-3 and caspase-7 (Vince et al., 2018). In our study, the expression of intrinsic apoptotic markers CASP-3 and BAX followed the same trend in the three conditions analyzed. We found a significant decrease of CASP3 expression in HL-1 + EVs-hGMSCs UN and in HL-1 + EVs-hGMSCs UH in comparison with control sample. In detail, CASP3 was found downregulated in HL-1 + EVs-hGMSCs UN in comparison with HL-1 + EVs-hGMSCs UH condition. BAX qPCR analysis, showed a downregulation in HL-1 + EVs-hGMSCs UN and in HL-1 + EVs-hGMSCs UH conditions in comparison with CTRL. These data suggest a potential regenerative role of EVs derived by hGMSCs, which seem able to reverse the apoptotic events induced by acute hypoxic exposure.

Despite the limitations related to an *in vitro* study, these findings collectively expanded our knowledge regarding EVs derived by hGMSCs and their potential protective and regenerative role against inflammatory and oxidative stress stimulus induced by acute hypoxia. Our study provides interesting results regarding the role of EVs derived by hGMSCs under acute hypoxic exposure that may affect cardiac tissue as candidates for novel perspectives in regenerative medicine.

Summarily the current work suggested the protective role against inflammatory and oxidative stress stimulus generated by acute hypoxia, modulating HIF-1α, P300, NRF2, NFkB, IL1B, CCL2, IL6, and the regenerative role through the VEGF signaling pathway and through the reversion of the apoptotic stimuli by the modulation of CASP3 and BAX.

Some molecular data discussed which led to the observed biological effects can be also supported from an epigenetic point of view.

Among the miRNAs identified within the EVs derived by hGMSCs used as treatment in our experimental model, some of these were linked with the gene pathways investigated leading to the observed effects on protein expression levels.

The overexpression of miR-138-5 significantly suppresses the expression level of HIF-1α (Bai et al., 2022). Additionally, it has been demonstrated the protective effect of miR-138-5 on cardiovascular damage due to the inhibition of aldosterone synthase (CYP11B2) leading to reversal of cardiac fibrotic remodeling (Kim et al., 2023). This miR-138-5p was detected within EVs-hGMSCs used could support the down-expression of HIF-1α that was detected trough confocal microscopy and Western blotting in HL-1 + EVs-hGMSCs UN and HL-1 + EVs-hGMSCs UH conditions. The same effect is mediated by miR-18a-5p which has been identified as inversely correlated with HIF expression levels (Krutilina et al., 2014). This miRNA is contained in the EVs h-GMSCs and could therefore epigenetically contribute to the HIF-α downregulation demonstrated in the present work. Based on the literature, miR-17-5p plays a cytoprotective role in response to hypoxia (Yu et al., 2020). This miRNA is contained in the EVs derived by hGMSCs, thus could contribute to the obtained protective effect against hypoxia stimulus.

In parallel, in the hypoxia pathway, among the miRNAs within the EVs h-GMSCs was found to be miR-150-5p interacting with p300, which from our data appears to be downregulated in the same manner of HIF-1α, being p300 its coactivator. It is well known that exists an inversely proportional correlation between miR-150-5p and p300, as previously reported by Li Z et al., which demonstrated that the upregulation of miR-150-5p resulted in the decrease of P300 expression levels such as the inhibition of miR-150-5p resulted in overexpression of p300 (Li et al., 2018). We can therefore highlight the target action of miR-150-5p on p300 expression, which may have contributed to the decreased p300 expression shown in the data from HL-1 + EVs-hGMSCs UN and HL-1 + EVs-hGMSCs UH conditions.

Regarding the typical inflammation marker NFkB, we identified a miRNA within EVs-hGMSCs that could be involved in the decreased expression of NFkB in the epigenetic control. miR-324-5p was seen to have a suppressing effect on NFkB activation. Previous researchers demonstrated that transfection with miR-21 mimics inhibited the expression of intra-nuclear NFkB p65 and downregulation of miR-21 significantly increased the activation of NFkB p65 (Zhu et al., 2018).

Therefore, these results demonstrated that miR-21 could block the activation of NFkB which is decreased HL-1 + EVs-hGMSCs UN and HL-1 + EVs-hGMSCs UH conditions. The same function is mediated by miR-324-5p which has been seen to be involved in blocking NFkB activation through CUE domain-containing 2 (CUEDC2) (Chi and Zhou, 2016). miR-324-5p was also found within the EVs analyzed. Our data report an increase expression of VEGF. One of the miRNAs that appears to interact with VEGF expression is miR-133a-3p. Previous works suggests that miR-133a-3p is able to inhibit cardiomyocyte apoptosis, and increases angiogenesis through upregulation of VEGF expression levels (Zhu et al., 2021). miR-133a-3p was found within the EVs-hGMSCs that may be involved in the overexpression of VEGF in the conditions treated with the EVs derived from h-GMSCs.

Another miRNA that interacts with VEGF expression, already explained above in EVs h-GMSCs, is miR-21-5p. Hu H. et al., demonstrated that exosomes derived from bone marrow mesenchymal stem cells promoted angiogenesis in an *in vivo* ischemic models through upregulation of miR-21-5p (Hu et al., 2022). Since this miRNA is contained in EVs derived from hGMSCs, we may hypnotized that it can act as a promoter of angiogenesis in our *in vitro* model.

Earlier work demonstrated that in an *in vitro model* of pneumocytes, exosomes derived from hMSCSs showed antioxidant capacities that attenuated apoptosis. This property was associated with the identification of miR-199a-5p in vesicles, which protect against oxidative stress by activating the NRF2 signaling pathway through the reduction of Caveolin1 (CAV1) gene expression leading to activation of the antioxidant signaling pathway NRF2(Gong et al., 2023). Based on this knowledge, the potential protective role against oxidative stress shown in the present work through the up-expression of NRF2 can be also liked to miR-199a-5p found inside the EVs h-GMSCs.

Then, NRF2 overexpression found in HL-1 + EVs-hGMSCs UN and HL-1 + EVs-hGMSCs UH conditions, may suggest a potential role of miR-199a-5p in the protection of oxidative stress reported in our study.

Within EVs-hGMSCs there are three miRNAs that could play a role in CASP3 downexpression observed in HL-1 + EVs-hGMSCs UN and HL-1 + EVs-hGMSCs UH conditions: miR-128-3p, miR-138-5p, and miR-221-3p. Liu S et al. demonstrated that in an *in vivo* mouse model of sepsis, induction of miR-128-3p reduced apoptosis and the production of pro-inflammatory factors in lung tissues. They saw that overexpression of miR-128-3p led to a large decrease in CASP3 activity (Liu et al., 2021). In an ischemia model, neuroprotection of astrocytes was observed following treatment with exosomes derived from bone marrow-derived mesenchymal stem cells overexpressing microRNA-138-5p. This has been demonstrated using miR-138-5p inhibitors. The results suggested a lower astrocyte migration rate after treatment with the miR-138-5p inhibitor, as well as treatment with the miR-138-5p mimic resulted in a decrease of CASP3 (Deng et al., 2019). Overexpression of miR-138-5p promotes proliferation and inhibits apoptosis. Sun L et al. demonstrated that overexpression of miR-221-3p using mimics in a cardiomyoblast cell line model and one of human umbilical vein endothelial cells resulted in down-expression of the proapoptotic protein, CASP3 as well as upregulation of miR-221-3p VEGF protein. These data indicated that miR-221-3p inhibits cardiomyocyte apoptosis and promotes angiogenesis (Sun et al., 2020). These information on miRNAs associated with CASP3 downexpression support the assumed involvement of these miRNAs contained in the studied EVs on CASP3 downexpression obtained in our experimental model. The epigenetic data described above lay the foundations for further studies on the potential role of the described miRNAs in the analyzed functions of different physio/pathological conditions affecting cardiomyocytes.

In conclusion, the EVs-hGMSCs may have a double effect: from one side they have shown a protective role under hypoxia due to the decrease of NFkB, IL1B, CCL2, IL6, HIF-1α, p300 and an increase of NFR2; while, from the other side they have shown a regenerative role under normoxia due to the increase of VEGF and the decrease of CASP3 and BAX. As the next stage research study should be directed to provide effective, safe, and powerful novel strategies based on the EVs derived from MSCs in the *in vivo* scenario.

## Data Availability

The raw data supporting the conclusion of this article will be made available by the authors, without undue reservation.
