# Supplementary figures and images for: Protective effect of oral stem cells extracellular vesicles on cardiomyocytes in hypoxia-reperfusion
Source: Front Cell Dev Biol. 2024 Jan 15;11:1260019. doi: 10.3389/fcell.2023.1260019 (PMC10823008; doi:10.3389/fcell.2023.1260019)

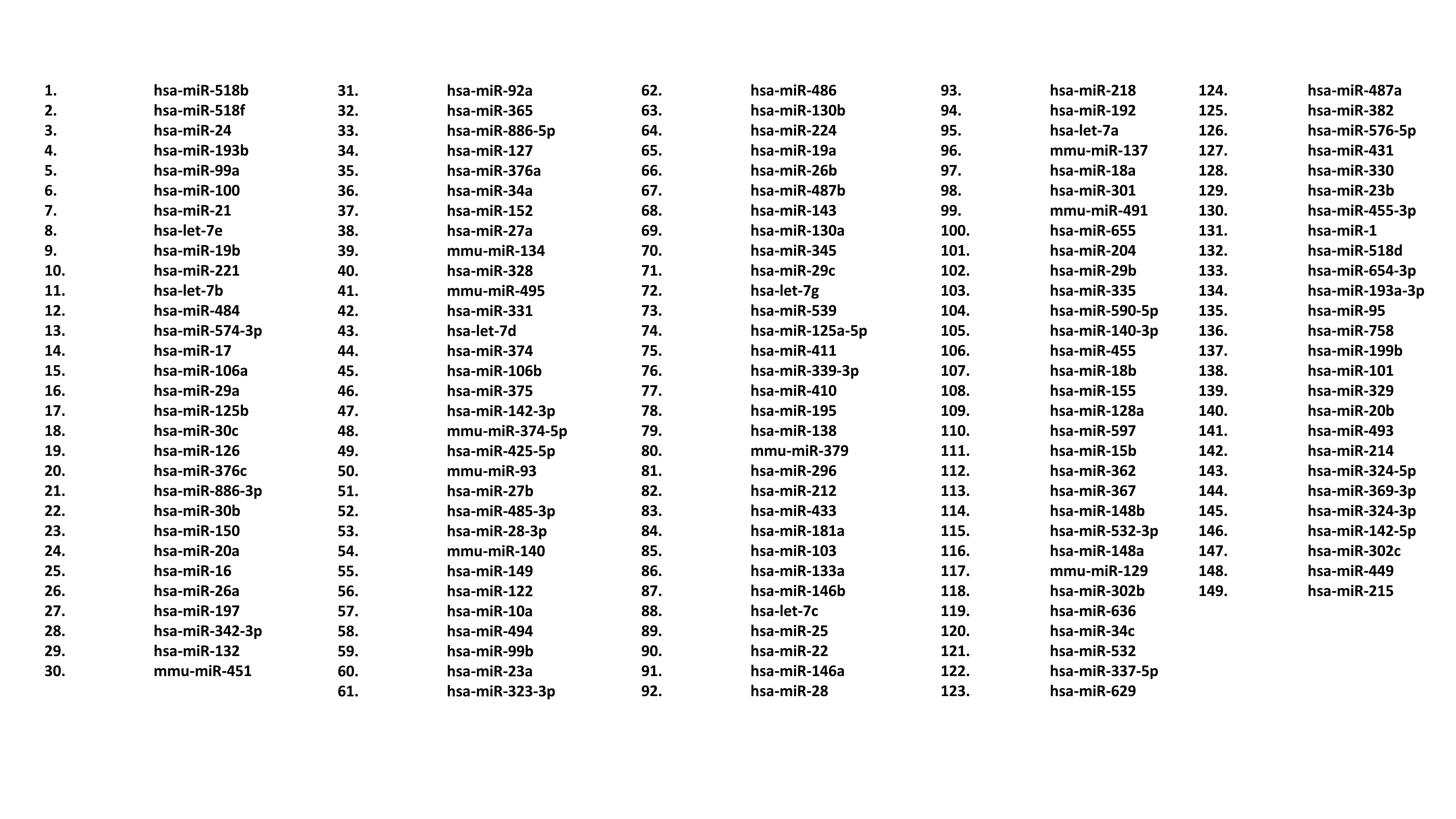

Supplement: Supplementary file 1 [file Image3.TIF]

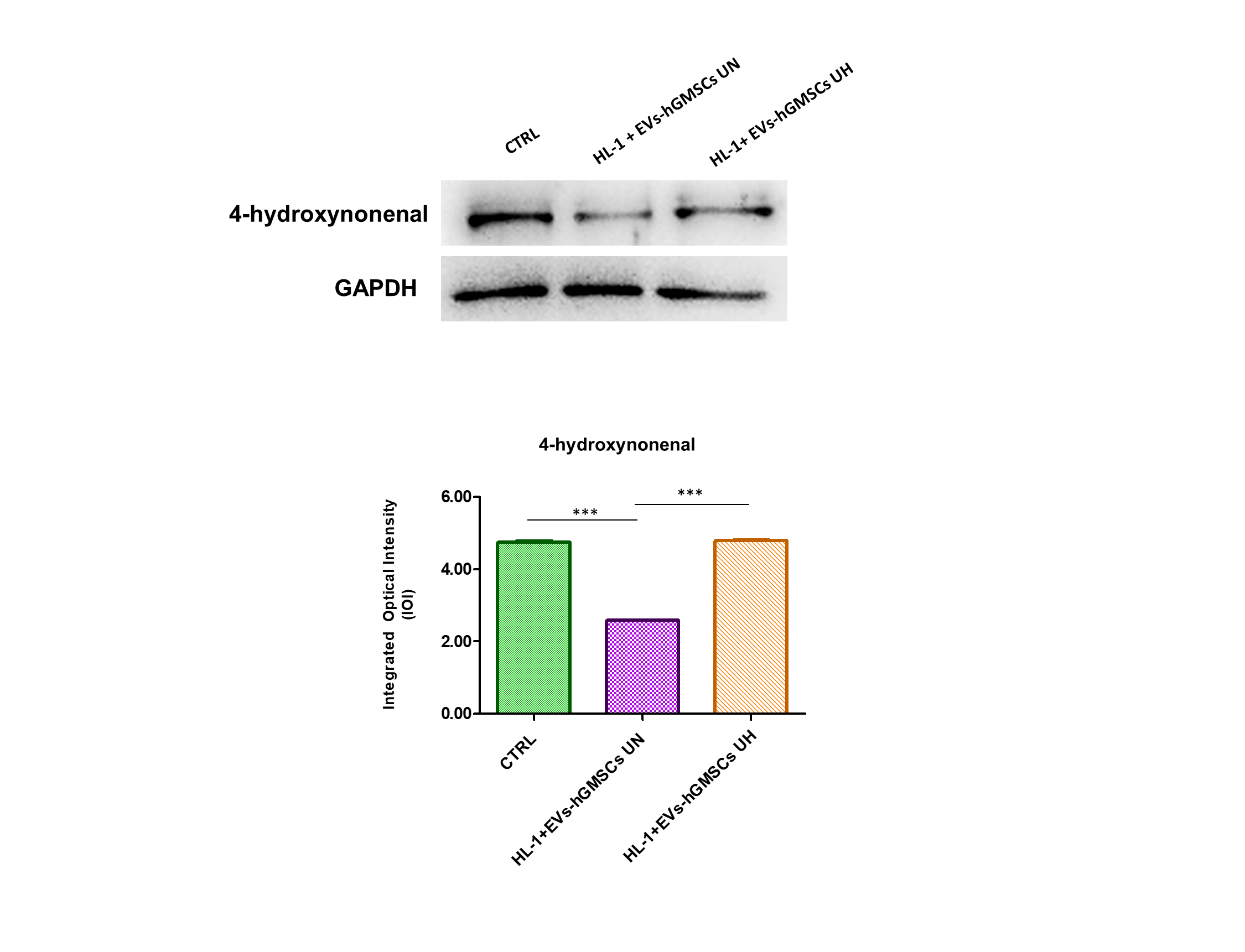

Supplement: Supplementary file 2 [file Image2.TIF]

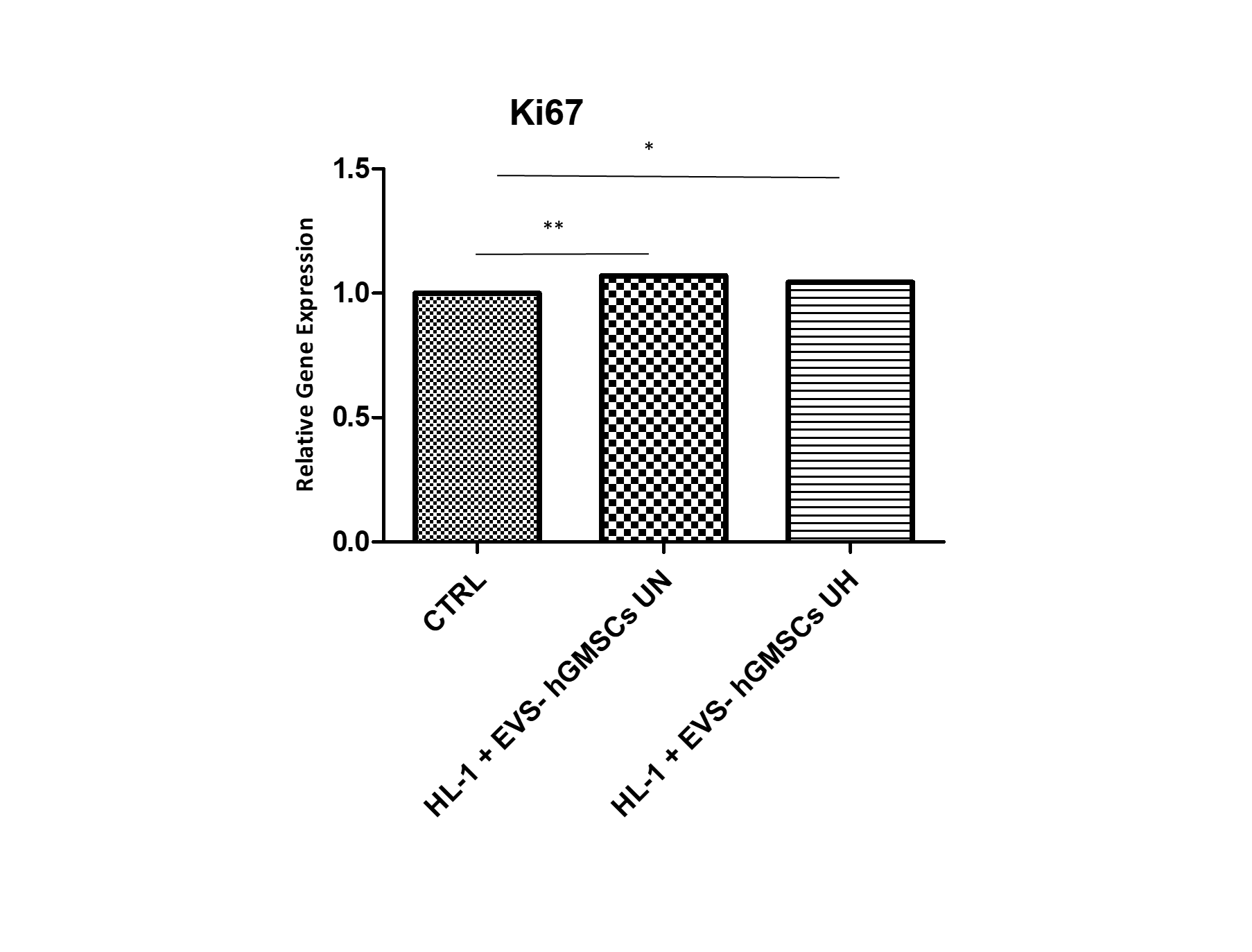

Supplement: Supplementary file 3 [file Image1.TIF]
